# Supplementary material for: Avidity of IgG to SARS-CoV-2 RBD as a Prognostic Factor for the Severity of COVID-19 Reinfection
Source: Viruses. 2022 Mar 16;14(3):617. doi: 10.3390/v14030617 (PMC8949074; doi:10.3390/v14030617)
Supplement: Supplementary file 1 [file viruses-14-00617-s001.zip › Supplementary file A_07-03-22.pdf]

Supplementary A. Dynamics for the IgG to RBD level and their avidity index along the time passed after immunization

Samples. 83 samples derived from healthy volunteers who were positive for IgG to the RBD of SARS-CoV-2 (positivity coefficient, PI ≥ 1,1 in ELISA) were studied. For each sample the date of previous immunization against SARS-CoV-2 was known. For reconvalescents (72 samples) this date was determined as the date of the first COVID-19 onset or as a date of the first positive PCR-test. For vaccinated (Sputnik V vaccine, 11 samples) the date of injection of the first dose of vaccine was set as the date of immunization. The information concerning the anamnesis of the immunization was based on the personal interview with the volunteers.

Methods. All the samples were tested for the IgG to RDB presence and their avidity index by the ELISA methods described in the main text of the paper. In statistical analysis, the Student t-criterion was used for comparison of the means, and binomial distribution was used to estimate confidence intervals for the proportion of qualitative characteristics in the groups.

Converting the ELISA results to BAU/ml. To make comparison of the data from different sources more convenient, we experimentally approximated the PC values gained form ELISA to the international units BAU/ml. For that, we build the calibration curve testing the dilutions of the NIBSC 20/136 standard (First WHO International Standard for anti-SARS-CoV-2 immunoglobulin, human[1]). The curve is shown at the Figure A-1. Because of pronounced linear shape of the curve, it became possible to estimate the formula for the PC to BAU/ml conversion:

BAU/ml = (PC+0,1396)/0.104 ≈ 10×PC + 1.3

We must strike, that the formula is just an estimation, because the used ELISA test was not certified as a quantitative, and during the routine runs no any calibrators were used except simple negative and positive controls (that is the reason why the results in the main part of the paper depicted in PC, not BAU/ml).

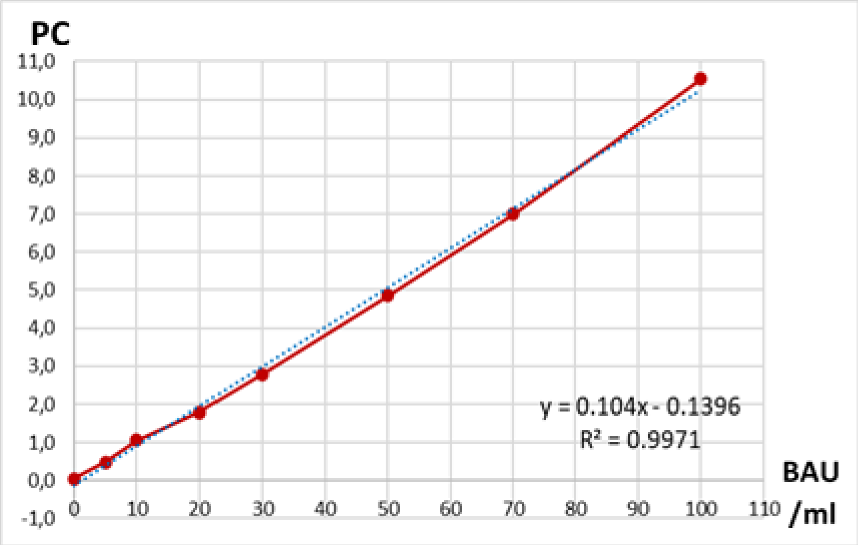

Figure A-S1. Calibration curve for the standard (NIBSC code: 20/136) in the used ELISA kit. The trend (dotted line), its function, and the correlation coefficient are depicted.

[1] Mattiuzzo et al. Establishment of the WHO International Standard and Reference Panel for anti-SARS-CoV-2 antibody: Expert Committee on Biological Standartization. Geneva, 9-10 December 2020. WHO/BS/2020.2403E

Dynamics of the IgG and their avidity with time since the immunization. The data for IgG quantity and the avidity index (AI) in the sera collected in different periods passed after the immunization are depicted at the Table A-1 and the Figure A-2. We found no significant differences for vaccinated and recovered after COVID-19 patients, so the results for them were joined. The obtained results suggest that on the third month after the immunization, a kind of seroconversion develops which generally decreases the IgG level, but in the same time the AI of these IgG increases (Figure A-2 B,D; Table A-1, lower part). When compare groups of patients who were immunized up to 2 months before the sample collection, and 3 and more months ago, one can see that the mean IgG level decreases from 100.3±16.2 to 58.3±12.5 BAU/ml (the difference is significant, $p<0.05$), and the mean AI increases from 33.2±5.6 до 57.7±5.1% ($p<0.05$). The patient on the third month are in the intermediate state: mean IgG and AI levels do not differ significantly from the groups of patients with ≤2 months or >3 months after the immunization (Figure 2-A, B,D). Thus, 3 months after the immunization is a transitional period form "low-avidity" to "high-avidity" immunity, and in may be suggested that the maturation of B-lymphocytes after the SARS-CoV-2 infection takes about 3 months.

Table A-S1. Data for mean IgG levels (in BAU/ml) and mean avidity index (AI, %) in the groups of patients formed by months after the immunization (the upper part of the table) or in the joined time groups (the lower part). CI: confidence interval of the mean (t-criterion, 95%).

Time passed after the immunization (up to the sample collection)	Mean IgG, BAU/ml	CI, BAU/ml (p<0.05)	Mean AI, %	CI of mean AI, % (p<0.05)	Number of samples, n (N=83)
**В разрезе месяцев**					
1 month	101.3	22.9	30.2	8.9	18
2 months	99.5	23.6	36.4	7.0	17
3 months	101.9	41.7	44.0	13.1	10
4 months	50.6	26.5	59.8	11.3	4
5 months	63.6	23.6	53.3	8.3	17
6 months	62.1	20.1	63.0	5.7	8
7 months and more	48.6	39.6	71.1	12.0	9
**В укрупненных группах**					
≤ 2 months	100.3	16.2	33.2	5.6	35
up to 3 months	101.9	41.7	44.0	13.1	10
>3 months	58.3	12.5	57.7	5.1	38

Discrimination of the cut-offs for interpretation of the "high-" and "low-avidity". At the Figure 2-A (D) one can see that AI in the patients (who, probably, have already finished the B-lymphocyte maturation after more than 3 months passed after the immunization), is upper than 50% (taking into account the lower border of the 95% CI). On the other hand, the AI in the patients who have just started the maturation, is reliable lower than 40% (Fig. 2-A, D). Thus, based on the mean AI in these two tome groups of patients (who have finished or not yet the maturation), the cut-offs were selected for the interpretation of the results: AI ≤40% means "low avidity", ≥50% – "high avidity", AI between 40 and 50% is "grey zone" (undetermined result, that required re-analysis of the patient serum in about one month after).

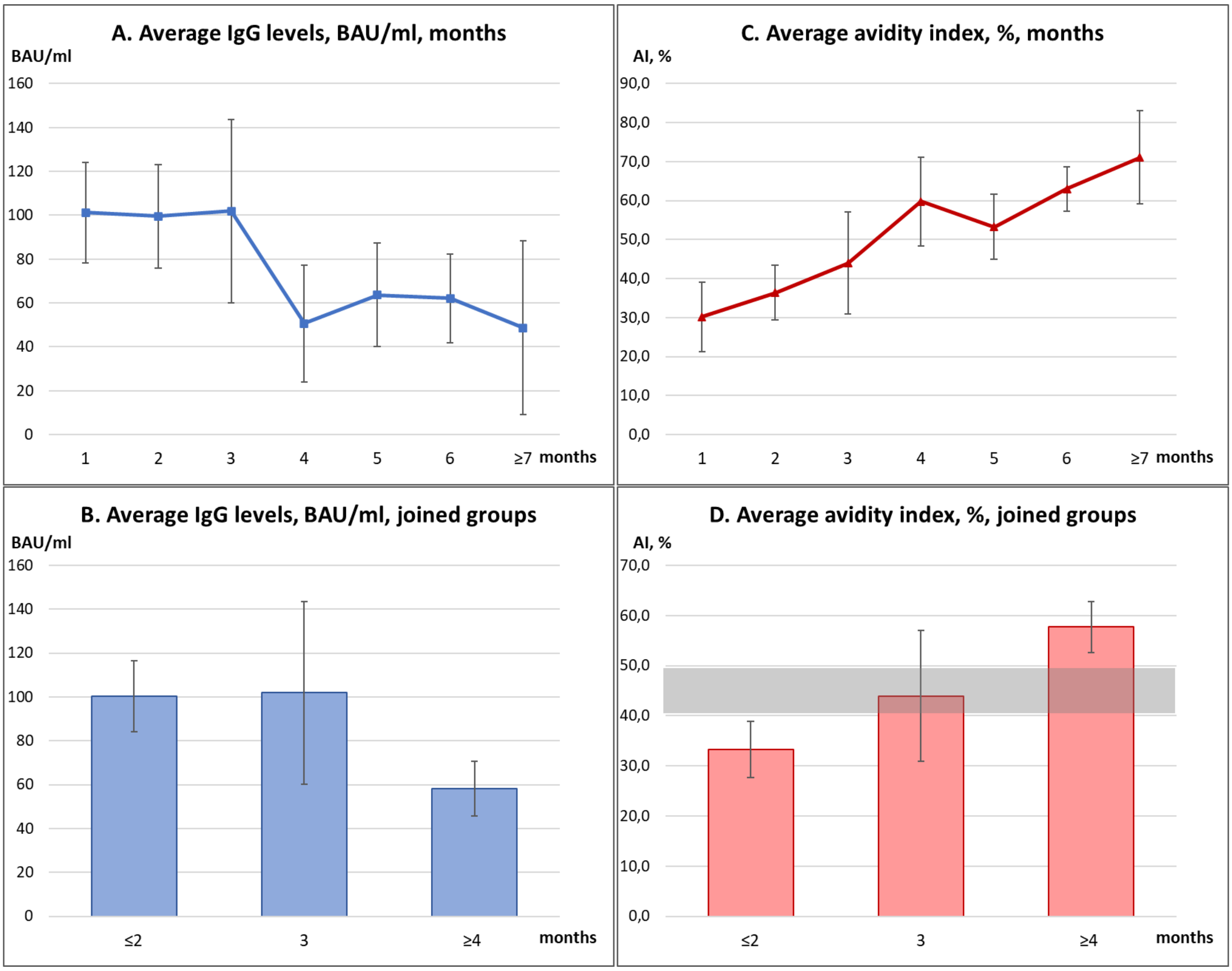

Figure A-S2. Mean IgG levels (BAU/ml) and avidity index (AI, %) by months after the immunization (A – mean IgG; C – mean AI), and in the join time groups (B,D). The numerical data are from the Table A-1. For each mean value the spread of the confidence interval is shown. The grey strip at the "D" graph discriminate the borders of AI levels: ≤40% means "low avidity", ≥50% – "high avidity", between 40 and 50% - "grey zone".

Conclusion: correlation between AI and the time of immunization (predictive ability). The proportions of "low avidity" (≤40%) and "high-avidity" (≥50%) IgG carriers differed significantly in the groups of patients who have completed or not the maturation (≤2 months and >3 months after immunization, respectively, Figure A-3). If we exclude the data with an undetermined interpretation (40%<AI<50%), and apply the Bayes theorem to others, we can count the probabilities of recent or longtime immunization depending on the AI we found in serum:

- if a patient carries the IgG of "low" avidity (AI≤40%), he or she was immunized in no more than 2 months ago with a chance of 88±11.5% (p<0.05);

- if a patient carries the IgG of "high" avidity (AI≥50%), he or she was immunized in more than 3 months ago with a chance of 91±10.1% (p<0.05).

At the time, 7.9±8.6% (p<0.05) of the patients who was immunized in more than 3 months ago, are still produce the IgG of "low" avidity (Figure A-3). It is reasonable to assume that in these patients the development of immunity has been impaired: B-lymphocyte maturation has not been completed and the patients have not acquired a stable immunity. Thus, they are at risk, the consequences of which in case of reinfection we describe in the main text of the article.

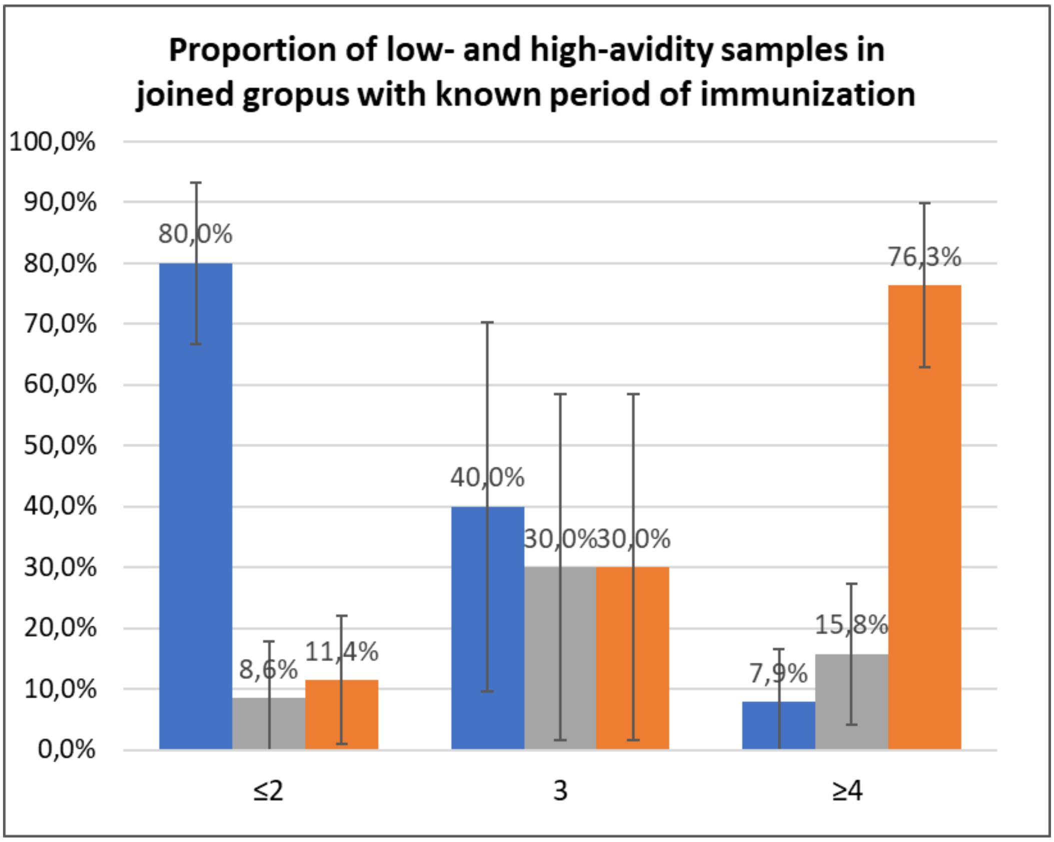

Figure A-S3. Proportions of the "low-avidity" (AI≤40%, blue) and "high-avidity" (AI≥50%, orange) carriers in the groups of volunteers with different periods of immunization. Grey columns – proportion of IgG carriers with undetermined result of AI (between 40 and 50%).
